# Remotely sensed between‐individual functional trait variation in a temperate forest

**DOI:** 10.1002/ece3.7758

**Published:** 2021-07-22

**Authors:** Carla Guillén‐Escribà, Fabian D. Schneider, Bernhard Schmid, Andrew Tedder, Felix Morsdorf, Reinhard Furrer, Andreas Hueni, Pascal A. Niklaus, Michael E. Schaepman

**Affiliations:** ^1^ Remote Sensing Laboratories Department of Geography University of Zürich Zürich Switzerland; ^2^ Jet Propulsion Laboratory California Institute of Technology Pasadena CA USA; ^3^ School of Chemistry and Biosciences Faculty of Life Sciences University of Bradford Bradford UK; ^4^ Department of Mathematics University of Zürich Zürich Switzerland; ^5^ Department of Computational Science University of Zürich Zürich Switzerland; ^6^ Department of Evolutionary Biology and Environmental Studies University of Zürich Zürich Switzerland; ^7^ Present address: Weesen Switzerland

**Keywords:** airborne imaging spectroscopy, airborne laser scanning, functional traits, phylogenetic variation, remote sensing, within‐species variation

## Abstract

1. Trait‐based ecology holds the promise to explain how plant communities work, for example, how functional diversity may support community productivity. However, so far it has been difficult to combine field‐based approaches assessing traits at the level of plant individuals with limited spatial coverage and approaches using remote sensing (RS) with complete spatial coverage but assessing traits at the level of vegetation pixels rather than individuals. By delineating all individual‐tree crowns within a temperate forest site and then assigning RS‐derived trait measures to these trees, we combine the two approaches, allowing us to use general linear models to estimate the influence of taxonomic or environmental variation on between‐ and within‐species variation across contiguous space.
2. We used airborne imaging spectroscopy and laser scanning to collect individual‐tree RS data from a mixed conifer‐angiosperm forest on a mountain slope extending over 5.5 ha and covering large environmental gradients in elevation as well as light and soil conditions. We derived three biochemical (leaf chlorophyll, carotenoids, and water content) and three architectural traits (plant area index, foliage‐height diversity, and canopy height), which had previously been used to characterize plant function, from the RS data. We then quantified the contributions of taxonomic and environmental variation and their interaction to trait variation and partitioned the remaining within‐species trait variation into smaller‐scale spatial and residual variation. We also investigated the correlation between functional trait and phylogenetic distances at the between‐species level. The forest consisted of 13 tree species of which eight occurred in sufficient abundance for quantitative analysis.
3. On average, taxonomic variation between species accounted for more than 15% of trait variation in biochemical traits but only around 5% (still highly significant) in architectural traits. Biochemical trait distances among species also showed a stronger correlation with phylogenetic distances than did architectural trait distances. Light and soil conditions together with elevation explained slightly more variation than taxonomy across all traits, but in particular increased plant area index (light) and reduced canopy height (elevation). Except for foliage‐height diversity, all traits were affected by significant interactions between taxonomic and environmental variation, the different responses of the eight species to the within‐site environmental gradients potentially contributing to the coexistence of the eight abundant species.
4. We conclude that with high‐resolution RS data it is possible to delineate individual‐tree crowns within a forest and thus assess functional traits derived from RS data at individual level. With this precondition fulfilled, it is then possible to apply tools commonly used in field‐based trait ecology to partition trait variation among individuals into taxonomic and potentially even genetic variation, environmental variation, and interactions between the two. The method proposed here presents a promising way of assessing individual‐based trait information with complete spatial coverage and thus allowing analysis of functional diversity at different scales. This information can help to better understand processes shaping community structure, productivity, and stability of forests.

## Graphical abstract

Trait‐based ecology holds the promise to explain how functional diversity may support community productivity. However, so far it has been difficult to combine field‐based and remote sensing (RS) approaches. By delineating all individual‐tree crowns within a temperate forest site and then assigning RS‐derived trait measures to these trees, we combine the two approaches, allowing us to estimate the influence of taxonomic or environmental variation on between‐ and within‐species variation across contiguous space.

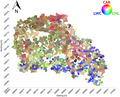

## 1 INTRODUCTION

Plant functional traits have specific spatial distributions as a result of different abiotic and biotic factors interacting at different spatial and temporal scales (Funk et al., 2017). For instance, environmental and local heterogeneity, phylogenetic distance, and plant–plant interactions such as competition and facilitation can act as important drivers of trait variation and affect coexistence mechanisms at different organizational levels from individuals to communities (Gross et al., 2009; Hart et al., 2016; Valladares et al., 2015). Understanding how these different drivers differ across spatial and temporal scales could help to gain insights into the possible responses of species, communities, and ecosystems to environmental change (Šímová et al., 2015).

Functional trait‐based approaches have been increasingly used to understand effects of trait variation on the assembly and functioning of communities and ecosystems (Cadotte & Davies, 2016; Díaz & Cabido, 2001; Kraft et al., 2015; Paine et al., 2011). For example, species coexistence is possible if intraspecific competition is weaker than interspecific competition, which according to limiting‐similarity theory can be achieved by larger trait differences between than within species (Macarthur & Levins, 1967). Generally, trait‐based approaches in community ecology have used species mean trait values (i.e., variation between different species, McGill et al., 2006), partly because they are available from the literature (Kattge et al., 2020). However, this approach may be biased by different local values of species mean traits and disregards the contribution of within‐species trait variation to the total trait variation. Within‐species trait variation can be large between sites due to large‐scale abiotic and biotic gradients (He et al., 2009) as well as within sites due to environmental heterogeneity and interactions between individuals (Auger & Shipley, 2013; Li et al., 2017; Roscher et al., 2018; Siefert et al., 2015) and due to genetic variation within species. Within‐species trait variation can affect community assembly and ecosystem functioning (Violle et al., 2012); however, to which extent it does so remains poorly investigated (Bongers et al., 2020). Here, we assess the within‐ and between‐species trait variation using remote sensing (RS).

Estimating between‐ and within‐species trait variation over spatially contiguous areas from the ground is extremely challenging (Duro et al., 2007), and therefore, most field studies either use sparse sampling (Li et al., 2017) or derive traits from the literature based on species lists. The sampling unit in such cases commonly is the individual plant, which represents a functional and genetic unit (Harper & White, 1974) with high ecological relevance. Remote sensing can provide continuous spatial coverage of optical and structural properties of vegetation canopies in a systematic and repeatable way and across different scales (Schimel et al., 2015; Skidmore et al., 2015). These optical and structural properties can be related to plant traits and thus be used to calculate functional diversity measures for vegetation (Schneider et al., 2017). In this case, the sampling unit is a pixel of vegetation in which different plant individuals and species can be mixed, thus making it difficult to estimate between‐ and within‐species trait variation. Attempts have been made to alleviate this problem by using small pixel sizes and then applying a “spectranomics approach” to link RS‐derived trait variation to taxonomic variation in tropical forest (Asner & Martin, 2011; Asner, Martin, Carranza‐Jiménez, et al., 2014; Chadwick & Asner, 2016; McManus et al., 2016). These studies have reported large contributions of between‐ relative to within‐species trait variation (for example Asner, Martin, Carranza‐Jiménez, et al., 2014 and Asner, Martin, Tupayachi, et al., 2014 quantified three times greater between‐species than within‐species variation for an extended set of biochemical traits). Nonetheless, without an explicit delineation of individuals, it remains difficult to assess this variation, not only because different individuals may still occur within a single pixel but also because single individuals may occur in multiple pixels.

Here, we combine the RS approach with an explicit delineation of individual‐tree crowns in a temperate forest (≈5.5 ha), allowing us to use general linear modeling as commonly done in ecology to partition trait variation among individuals into variation due to taxonomy (e.g., class, species) and environment. Our goal is to assess the potential of RS‐derived traits to identify taxonomic units. Traits with high taxonomic and low environmental variation between individuals would be promising tools to separate species and by extension even genotypes within species in the future (Cavender‐Bares et al., 2016). Furthermore, we aim to describe environmental influences on trait variation between and within species for a complete forest stand. With the mapping of individual‐tree traits, we want to bridge the gap between field and remote sensing measurements, and to open doors to the possibility of studying biodiversity at a very high spatial resolution under natural conditions. Through our measurements, we specifically ask: (a) Can plant traits be assessed by remote sensing at the level of individual‐tree crowns? (b) To which extent can different traits be used to identify and map taxonomic units? (c) How does environmental variation influence variation in these traits and does this vary among taxonomic units?

## 2 MATERIALS

### 2.1 Study area

**FIGURE 1:**
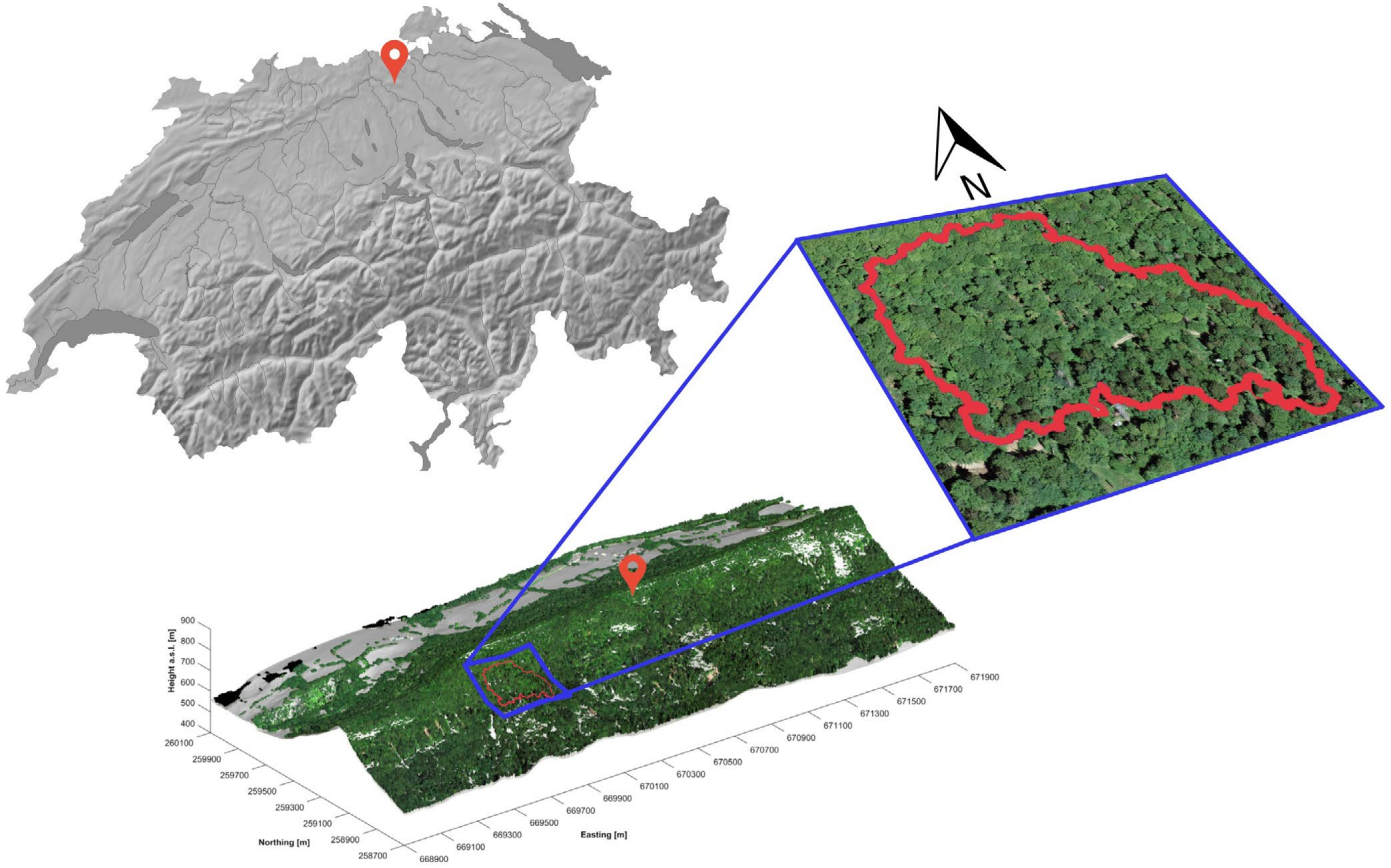
Geographic location of the study site. Top left: Red map pointer indicates the location of Lägern Mountain in Switzerland. Bottom: 3D representation of the study area and zoom of the 5.5 ha plot site

Lägern Mountain is a seminatural mixed temperate forest located approximately 15 km northwest of Zürich, Switzerland (Eugster et al., 2007). This mountain lies on the eastern part of the Jura foothills, where the climate is humid (mean annual precipitation of 1,000 mm) and mild (mean annual air temperature of 7.4°C) (Etzold et al., 2011). Our study includes RS data and in situ measurements from a nonmanaged 5.5‐ha forest plot (47°28′N, 8°21′E), situated between 620 and 810 m a.s.l. on the steep south‐facing slope (with some parts up to 60°) of the Lägern Mountain (Figure 1).

The plot is one of the six research sites of the University Research Priority Program (URPP) on Global Change and Biodiversity of the University of Zürich and includes 1,307 canopy trees with a diameter at breast height (DBH) >20 cm and ranging in age from 52 to 185 years (Eugster et al., 2007; Morsdorf et al., 2020). The trees belong to three conifer and 10 angiosperm species. Dominant species are European beech (*Fagus sylvatica*), ash (*Fraxinus excelsior*), and sycamore (*Acer pseudoplatanus*), which together with the other species, that is, European silver fir (*Abies alba*), Norway spruce (*Picea abies*), Scots pine (*Pinus sylvestris*), Norway maple (*Acer platanoides*), field maple (*Acer campestre*), European hornbeam (*Carpinus betulus*), sessile oak (*Quercus petraea*), whitebeam (*Sorbus aria*), wych elm (*Ulmus glabra*), and large‐leaved lime (*Tilia plathypyllos*) shape a complex forest structure over a number of canopy layers (Schneider et al., 2014).

### 2.2 Field data

Leiterer et al. (2015) collected individual‐tree data from the plot during an extensive field campaign, in which all trees with DBH >20 cm were inventoried. For each tree, various architectural and spatial parameters such as DBH, crown dimensions, social status regarding neighboring trees, and tree position were measured. In addition, forest experts identified for each of the trees the species name, interpreted the canopy complexity, and described the soil properties on the tree location such as soil type, depth, and grain size, among others. Table 1 shows taxonomic information of the test site (corresponding class, family, and genus of each species), total and relative abundance of species, and all in situ measured spatial and architectural variables for each individual tree.

**TABLE 1:** Information of the individual trees and parameters measured during the field campaign

Class	Family	Genus	Species	Total number individuals (% of total individuals)	In situ measured spatial and structural parameters for each of the 1,307 individual trees
A	Fagaceae	*Fagus*	*sylvatica*	518 (39.6)	Tree position
A	Oleaceae	*Fraxinus*	*excelsior*	252 (19.3)	Species name
A	Sapindaceae	*Acer*	*pseudoplatanus*	168 (12.9)	DBH
C	Pinaceae	*Abies*	*alba*	108 (8.3)	Type of canopy layering
A	Malvaceae	*Tilia*	*platyphyllos*	105 (8.0)	Crown dimensions
C	Pinaceae	*Picea*	*abies*	51 (4.0)	Crown shift regarding trunk position
A	Ulmaceae	*Ulmus*	*glabra*	43 (3.3)	Social dominance
A	Sapindaceae	*Acer*	*platanoides*	40 (3.0)	Presence and type of understory layer
A	Fagaceae	*Quercus*	*petrea*	9 (0.7)	Presence and type of herbs layer
A	Betulaceae	*Carpinus*	*betulus*	8 (0.6)	Soil type
A	Sapindaceae	*Acer*	*campestre*	3 (0.2)	Soil depth
C	Pinaceae	*Pinus*	*sylvestris*	1 (0.1)	Soil rocks
A	Rosaceae	*Sorbus*	*aria*	1 (0.1)	Soil grain size
	Total families: 8	Total genera: 11	Total species: 13	Total individuals: 1,307	

Note

Species excluded from statistical analyses because small sample sizes are highlighted in gray.

Abbreviations: A, angiosperms; C, conifers.

### 2.3 Airborne RS data

Different RS techniques such as airborne imaging spectroscopy (AIS) and airborne laser scanning (ALS) have been employed to assess biochemical and architectural functional diversity of forests. On the one hand, AIS offers high spectral resolution data, useful for discriminating subtle variations in spectral properties of vegetated surfaces (Schaepman et al., 2009; Schneider et al., 2017). These variations can be influenced by changes in the architectural and chemical properties of tree canopies (Ustin & Gamon, 2010). On the other hand, ALS can actively retrieve horizontal and vertical 3D architectural information of forest canopies (Morsdorf et al., 2009; Wulder et al., 2012). Successful attempts have been made to retrieve biochemical and architectural trait information from tree canopies using AIS and ALS (Asner & Martin, 2009; Kokaly et al., 2009; Ustin et al., 2009). The combined use of AIS and ALS data can build unique high‐resolution functional trait datasets, allowing an accurate characterization of canopy biochemistry and architecture (Asner et al., 2012; Torabzadeh et al., 2015). AIS information was acquired by the Airborne Prism Experiment (APEX) imaging spectrometer (Schaepman et al., 2015) on 17 July 2016 between 12:13 UTC and 12:35 UTC under clear sky conditions. The data were collected from an altitude of 4,540 m a.s.l. in 316 spectral bands ranging from 372 to 2,506 nm and resulting in a ground pixel size of 2 m. The raw APEX data stream was processed to calibrate radiances in the APEX Processing and Archiving Facility (Hueni et al., 2009, 2012). The processing included the estimation of spectral shifts and misregistration (smile) by the ATCOR smile module using atmospheric absorption features (Richter et al., 2011) and the consequent compensation of spectral shift‐related radiometric biases due to the beam‐splitting coating in the APEX optical subunit (Hueni et al., 2014). The spectro‐radiometric calibration was based on coefficients generated by the APEX Calibration Information system (Hueni et al., 2013), applying individual radiometric gains and offsets for all spatio‐spectral pixels and correcting bad pixels and wire‐positions by linear interpolation (Jehle et al., 2010). Radiance data were then converted to reflectance factors with uniform center wavelengths by ATCOR (Hueni et al., 2017; Richter & Schläpfer, 2002) with the topographic information obtained through parametric geo‐coding by PARGE (Schläpfer & Richter, 2002), based on the swisstopo swissALTI3D digital elevation model. The final 284 spectral bands of the reflectance product covered 400–2,424 nm after removing overlaps between VNIR and SWIR detectors and noisy bands at the spectral edges of the two channels.

Airborne laser scanning data were acquired by two helicopter‐based laser scanning campaigns carried out on 10 April and 1 August 2010 under leaf‐off and leaf‐on conditions, respectively. Measurements were taken with a RIEGLs LMS‐Q680i (max.nominal scan angle ±15°) at a mean flying altitude of 500 m above ground. Data were acquired in strips with an overlap of 50%, and average point density was 40 pts/m^2^ (from a pulse repetition rate of 200 kHz). The leaf‐on dataset was used to retrieve architectural information of individual trees. Leaf‐off data were used to derive the Digital Terrain Model (DTM) which was retrieved by an interpolation using an algorithm presented in Leiterer et al. (2013).

The fact that AIS and ALS datasets were retrieved in two different years (2016 for AIS data and 2010 for ALS data) should not entail major problems. Tree architecture in our test site is considered mostly stable during the last 6 years because our study focuses on dominant trees and no extreme meteorological phenomena nor anthropogenic disturbances occurred during this period.

### 2.4 Environmental variables

Aspect, curvature, slope, and elevation were calculated from the DTM derived from the ALS campaigns in 2010 under leaf‐off conditions (Leiterer et al., 2013). Then, to assign specific topographic values to individual trees, the median for categorical variables such as aspect and the mean for the rest of the variables were taken from all the pixels inside each polygon representing an individual tree. Soil data (soil type, depth, and volume of coarse grain) were acquired from Bodenkarte Baden (Landeskarte der Schweiz 1:25,000) provided by Eidgenössische Forschungsanstalt für Agrarökologie und Landbau (FAL). Incoming photosynthetically active radiation (PAR) was simulated for the whole site using the radiative transfer model DART (Schneider et al., 2017; Appendix 22). Table 2 lists the environmental variables and their ranges across the study site.

**TABLE 2:** Environmental variables and their means and ranges across the study site

Variable	Unit	Mean	Range
PAR	W m^−2^ day^−1^	3,919	525, 8,414
Elevation	m	724	655, 810
Aspect	Categorical	—	N, NE, E, SE, S, SW, W, NW
Slope	Degrees	30.8	3.9, 58.4
Curvature	—	0.0041	−0.387, 0.287
Soil type	Categorical	—	Cambisol, calcic cambisols, leptosols
Soil depth	Categorical	—	30−50 cm, 50−70 cm, 70−100 cm
Soil rocks	Categorical	—	<10 vol. %, 10–30 vol. %, >30 vol. %
Understory	Categorical	—	None, limestone shrubs, elder, hazel, holly, coniferous young forest broadleaf young forest, mixed, others
Herb layer	Categorical	—	None, spring flowering, perennial grass, blackberry, evergreen ground cover coniferous young forest, broadleaf young forest, mixed, others

### 2.5 Phylogenetic data

**FIGURE 2:**
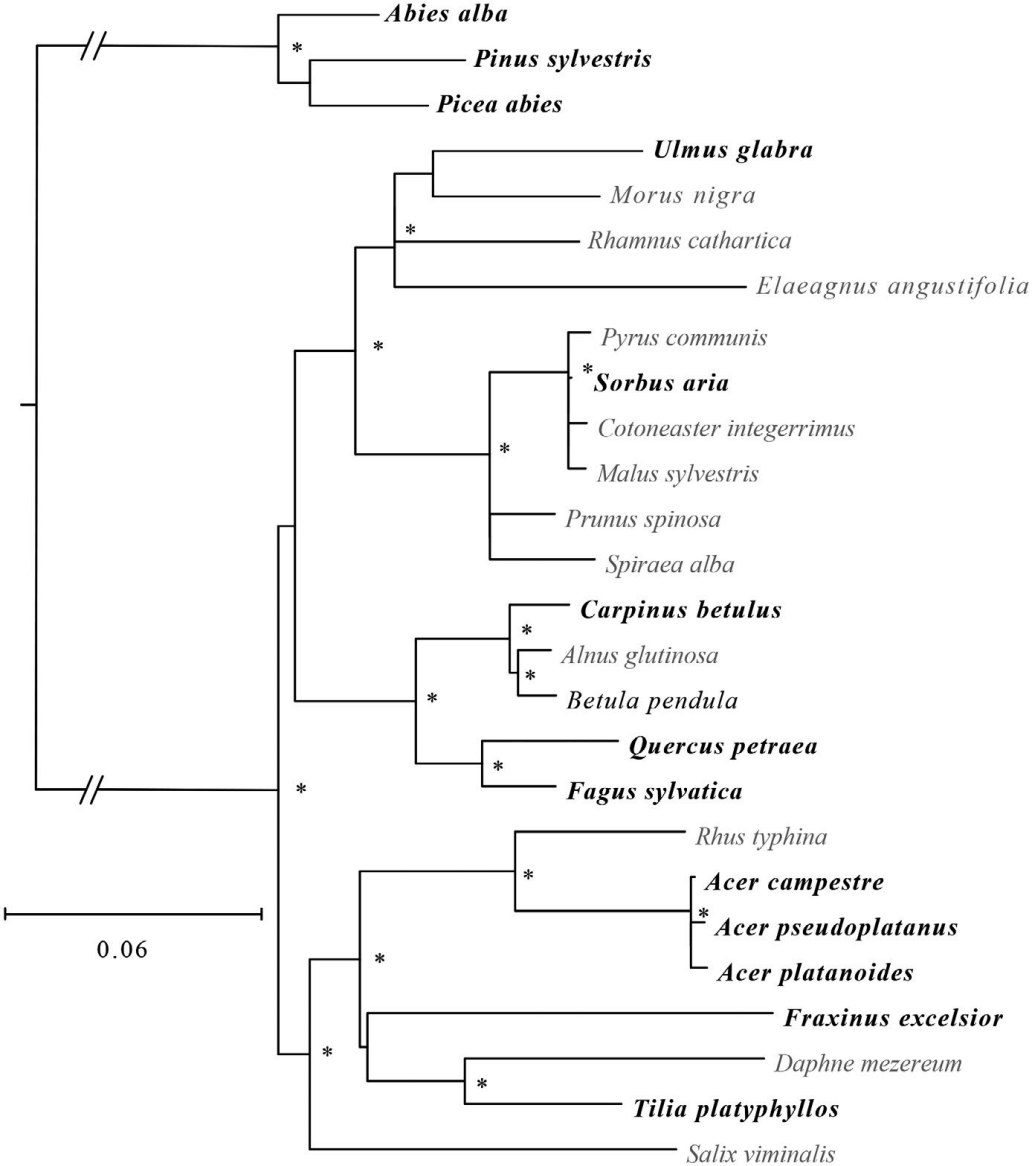
Phylogenetic tree including the 13 species of the test site (in bold). * represents bootstrap support above 70%

To assess the phylogenetic relationship between the 13 tree species, we used an in‐house Python script to automate the retrieval of sequence information for two commonly sequenced genes, *rbcL* and *matK*, from GenBank (Benson, 2004; Appendix 7). Because the importance of taxon sampling has been well documented to influence the resolution of phylogenies (Nabhan & Sarkar, 2012), we also included 13 further European woody herb and tree species identified from the DaPhnE phylogeny (Durka & Michalski, 2012; Appendix 7). Trimmed sequences for each gene were concatenated and then aligned using MAFFT (Katoh & Standley, 2013), and assessed visually for misalignments in SeaView v4 (Gouy et al., 2010). We then used RAxML v8 (Stamatakis, 2014) to generate a maximum likelihood tree with 1,000 bootstrap replicates. The resulting tree (Figure 2) was then rooted using the three conifer species as an outgroup. A distance matrix between species was computed using DNADIST in PHYLIP v3.6 (Felsenstein, 2005) with the F84 model. Confidence intervals were computed for the distance matrix using 1,000 random datasets generated using SEQBOOT.

## 3 METHODS

### 3.1 Individual tree crown mapping

**FIGURE 3:**
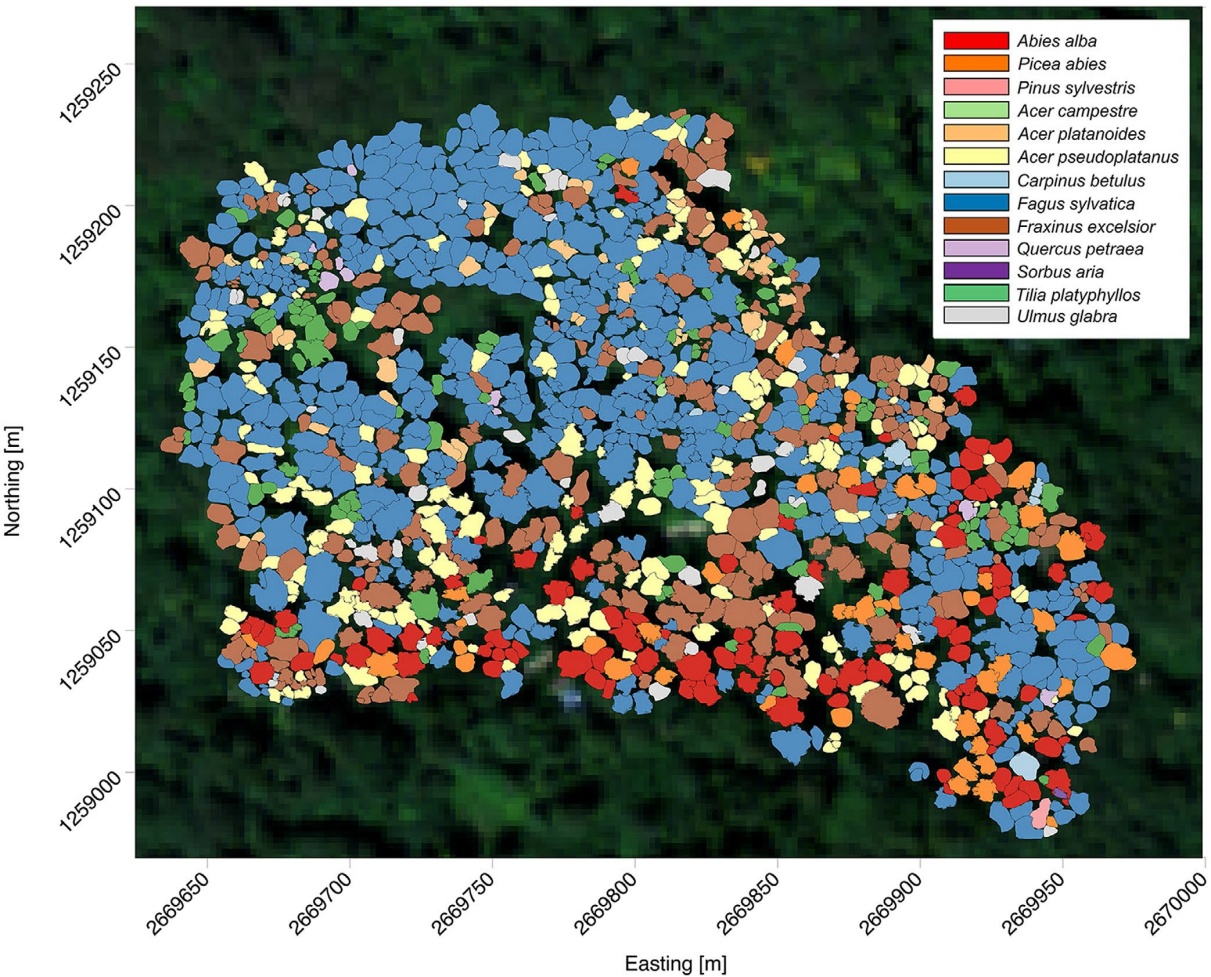
Individual tree crown map. Geographic location of individual tree crowns in the 5.5 ha plot site. Each tree species is represented by a different color. Axis shows coordinates expressed in the CH1903 LV03 Swiss coordinate system

The Lägern test site is located in an area with steep slopes, causing many of the tree trunks to grow tilted. This condition can complicate the matching of RS data with ground truth, as measured stem positions may be offset to crown centroids. ALS data can provide individual‐tree crown information. However, in mixed and complex forests such as Lägern, detection accuracy may be as low as 50%, and it is best to complement different datasets, for example, ALS with ground truth data (Wang et al., 2016). Here, stem positions were matched with crown segments obtained with a semiautomatic segmentation approach (Haara & Haarala, 2002) that was applied to different datasets such as ground truth information, UAV‐detected individual‐tree crown boundaries (ITCs), and individual‐tree segments from ALS point clouds. Ground‐truth data were acquired during a field campaign using tachymeter and visual inspection, as presented in Leiterer et al. (2015), and included information such as coordinates, DBH, social status, approximate crown size, and horizontal crown displacement of each tree at the test site. Individual tree crown boundaries were detected from combining a UAV‐derived ortho‐mosaic (flights were performed during the fall of 2013) and a ALS‐derived canopy height model (Torabzadeh et al., 2014, 2019). Individual tree segments from ALS were derived with the approach of Parkan and Tuia (2015) using geodesic voting. Ground‐truthed tree positions were linked to ITCs and geodesing voting‐derived point clouds using coordinates, crown size, and vertical layering as main constraints. Finally, overlaps and unassigned crowns were visually matched and corrected by further field visits by the authors in 2016. A total of 1,307 trees from 13 different species were mapped and used as the reference dataset (Figure 3).

### 3.2 Functional traits retrieval from airborne RS data

#### 3.2.1 Biochemical traits

Three biochemical and three architectural functional traits were retrieved from AIS and ALS data, respectively, for each individual tree. The selection of traits was based on their ecological relevance and feasibility to be measured by AIS and ALS. Relative content of chlorophylls (CHL) and carotenoids (CAR), and relative leaf water content (LWC) were the three traits used to assess the biochemical composition of tree crowns. Pigments such as chlorophylls are involved in the process of light harvesting and conversion of the energy into glucose and other products (Krause & Weis, 1991), and carotenoids are essential in providing photoprotection to chlorophylls in the presence of oxygen (Moore et al., 1989). Because leaf pigments play a major role in photosynthesis and leaf protection, assessing pigment content could give insights into the leaf functioning of individual trees (Ustin et al., 2009).

To collect this information, spectral reflectance signatures were retrieved from individual sun‐illuminated canopies. A shadow mask was applied on the spectral data to remove pixels that could lie in shade and induce weakening of the spectral signal, and consequently increase the spectral variability. Since the resolution of spectral data was 2 m, we selected all pixels lying within each crown boundary using pixel centroids. We calculated mean reflectance for each spectral wavelength for each crown. Pigment relative content (CHL and CAR) and LWC were then calculated following the formulae presented in Appendix 1. Leaf‐level chlorophyll estimates were compared with relative chlorophyll content of modeled canopy spectra (Appendix 3), and all canopy pigment and leaf water content estimates for each of the individual trees in our test site were compared against 2010 data from the same trees used in Schneider et al. (2017), see Appendix 4.

#### 3.2.2 Architectural traits

The assessment of individual‐tree architecture can provide functional traits related to light interception and growth, that is, traits that might have an impact on important ecosystem services such as carbon storage and biomass supply (Singh et al., 2015). The three architectural traits selected in this study were canopy height (CH), plant area index (PAI), and foliage‐height diversity (FHD). FHD was derived for canopy layers of 5 m. Detailed explanations and formulae used to calculate these three traits are provided in Appendix 2, and use across different spatial scales and light detection and ranging (lidar) techniques are demonstrated in Schneider et al. (2020, 2019, 2014).

Overall normality of data and colinearity were assessed by frequency distributions and Pearson pairwise correlations both for biochemical and architectural traits (Appendices 5 and 6). Note, however, that for the general linear analyses explained below only normality of residuals was required.

### 3.3 Data analysis

#### 3.3.1 Functional trait variation map

**FIGURE 4:**
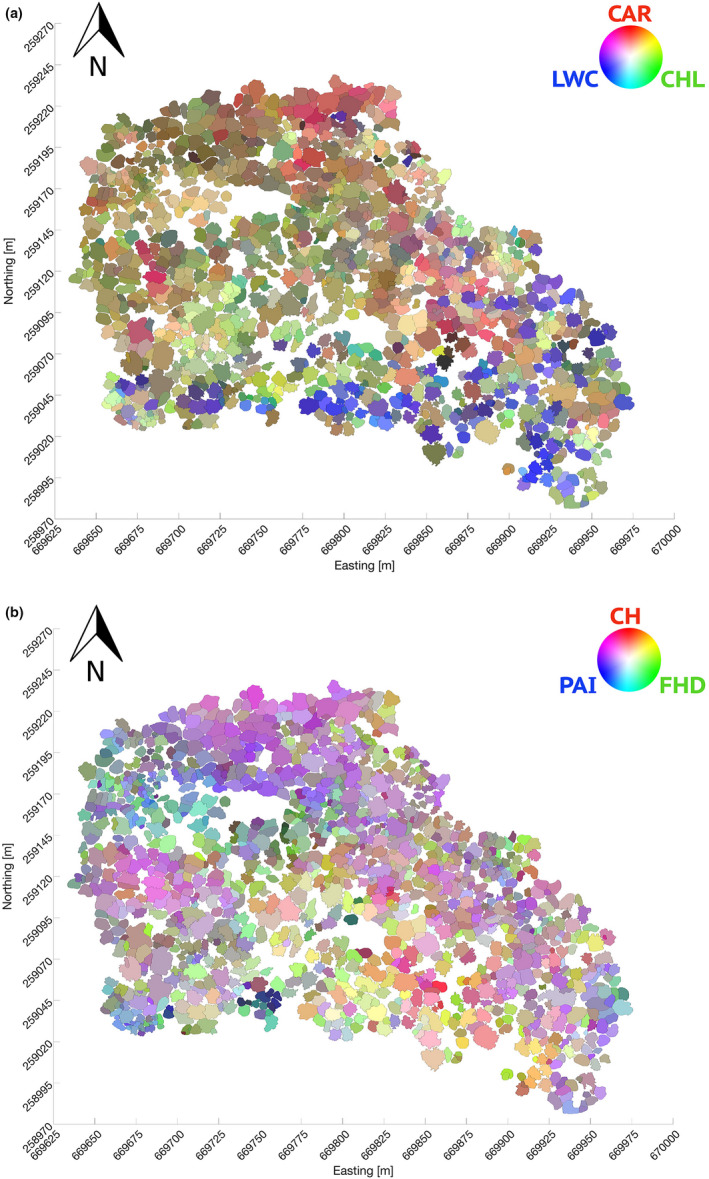
Maps of functional trait variation of the Lägern temperate forest test site. Individual crowns are characterized using (a) an RGB color composite of three biochemical traits (CHL, CAR and LWC: brightest green highest relative CHL content, brightest red highest relative CAR content and brightest blue highest LWC) and (b) using an RGB color composite of three architectural traits (PAI, FHD, and CH: brightest green highest FHD, brightest red highest CH, and brightest blue highest PAI). Axis show coordinates expressed in the CH1903 LV03 Swiss coordinate system.

To map functional trait variation at the Lägern temperate forest test site, each individual tree was characterized by its unique biochemical and architectural fingerprint following the already presented methods for trait retrieval. To this end, we grouped the three biochemical (CHL, CAR, LWC) traits in one map and the three architectural traits (PAI, FHD, CH) in another map. In each of these maps, the values of the three traits were assigned to an RGB color resulting in two final crown color maps (Figure 4).

#### 3.3.2 Assigning individuals to species using DA

We used discriminant analysis as implemented with the function lda() in R (R Core Team, 2016) to test how well tree individuals could be assigned to their species as identified on the ground using classification functions calculated with the six traits.

#### 3.3.3 Relation between phylogenetic distances and trait and spectral distances

To assess how the phylogenetic distances between the eight most abundant species (the remaining five had too few individuals to be included in the analysis) at the site (Figure 2) were related to trait and spectral distances, Mantel tests were conducted using the “mantel.rtest” function from the R (version 3.2.1) package ade4. Correlation coefficients (*r*) and *p*‐values were determined by 9,999 permutations. Three different trait distance matrices were computed using three different groups of traits: all six traits together, the three biochemical traits, and the three architectural traits. Trait distances were calculated as Euclidean distances from data rescaled to 0–1 (minimum–maximum). Spectral distance matrices were calculated using all spectral information from the 284 spectral bands in the visible to short‐wave infrared spectral range (400–2,400 nm). All trait, phylogenetic, and spectral distances were rescaled using a linear transformation on the raw data to a value range of 0–1, with 0 and 1 being the lowest and highest distance of the dataset, respectively. We also calculated trait distances using predicted trait values for the eight species under the same mean environment after general linear model analysis (see below). However, because results were very similar to those with species traits not corrected in this way for environmental variation, we do not report these results.

#### 3.3.4 Analysis of trait variation using general linear models

We used general linear modeling followed by analysis of variance (ANOVA) to assess the influence of taxonomy and environmental variation on the six RS‐derived functional traits (“aov” function in R package “stats,” version 3.3.0, R Core Team, 2016). We sequentially fitted first the taxonomic terms class and species (note that these can be added to an overall species term), then the environmental terms such as soil type, soil rocks, soil depth, PAR, aspect, elevation, slope, curvature, herbs, and understory (see Table 2; in sum an overall environment term), and then the interaction between taxonomy and environment and finally two spatial grids G90 and G40, accounting for spatial variation not explained by the previous taxonomic and environmental terms (Appendix 22). Terms that were not significant individually were removed from final models (Appendices 8, 9, 10, 11, 12). Subsequently, we aggregated terms for variance partitioning as displayed in Figure 7. We used the percentage sum of square explained by the different terms to partition the total variance among individuals into variation between species (taxonomic terms) and within species (environmental terms and residual). The residual within‐species variation could have been due to genetic variation within species, small‐scale environmental variation, or measurement errors. Note that this approach to partition variance differs from the one used for example by Asner and Martin (2011). Whereas they used random‐effects models and thus estimated variance components (VC) for each explanatory term, we used fixed effects and thus estimated sum of squares (*SS*). These are increments in multiple *R* ^2^ and have the advantage that they are additive. For example, if class explains 2% of total *SS* and species 4%, then together they explain 6%. However, if the VC for class is 2 and the VC for species is 4, then the VC for the two combined will typically not be 6, but rather <6. Thus, using VCs in a figure like Figure 7 can be misleading (Green & Tukey, 1960). Finally, we combined the six traits in a multivariate ANOVA (MANOVA) to assess whether multivariate trait variation showed clearer taxonomic differences (which it did not, see Appendix 14 and Section 4).

## 4 RESULTS

### 4.1 Traits assessed by remote sensing at the level of individual‐tree crowns

In answer to our first question in Section 1, it was possible to assess traits at the level of individual‐tree crowns, once these were delineated (Figure 2). In Figure 4a, individual crowns from the southern part of the test site, where the majority of conifer species are found (Figure 3), have higher LWC values than crowns from the northern part, mostly dominated by angiosperms (with a high presence of *Fa. sylvatica* individuals). For pigments, there is a tendency of higher CAR contents on the eastern side and higher CHL contents on the mid‐southern side of the site. Although individual‐tree crown values do not show a strong trend along the spatial gradient, a general tendency of more complex canopy layering (higher FHD) on the south and denser canopies (higher PAI) on the north can be perceived. Canopy height (CH) does not present a clear spatial pattern, but there are some local patches with higher trees in the northern and mid‐western part and smaller trees in the north‐western part.

### 4.2 Between‐species trait variation

Our second question in Section 1 was to which extent the RS‐derived trait variation among individual trees could be used to identify and map taxonomic units. Using all traits in a MANOVA (Appendix 14), only 3.5% of the total variation among individuals could be assigned to differences between taxonomic units, and of these, only 0.1% were due to differences between the two classes: gymnosperms and angiosperms. In contrast, individual biochemical traits allowed a much better discrimination between the two classes, which were explaining 10.6%, 15.7%, and 26.3% of the variation in CHL, CAR, and LWC, respectively (Appendices 8, 9, 10). On average, the two conifer species had lower levels of CHL and CAR and higher levels of LWC than the six angiosperm species (Appendices 15, 16, 17). Additional differences between species within the two classes were still highly significant but contributing less variation, namely 5.2%, 2.9%, and 3.9%, respectively, in CHL, CAR, and LWC (Appendices 8, 9, 10). For the architectural traits, variation between taxonomic units was similarly low as in the MANOVA, with class and species together explaining 3.1%, 4.0%, and 6.6% of the variation in FHD, PAI, and CH, respectively (Appendices 11, 12, 13). Using all six traits in a discriminant analysis, which maximizes the potential to assign individuals to the predefined taxa, correctly assigned 49% of all individuals to species (Appendix 21).

**FIGURE 5:**
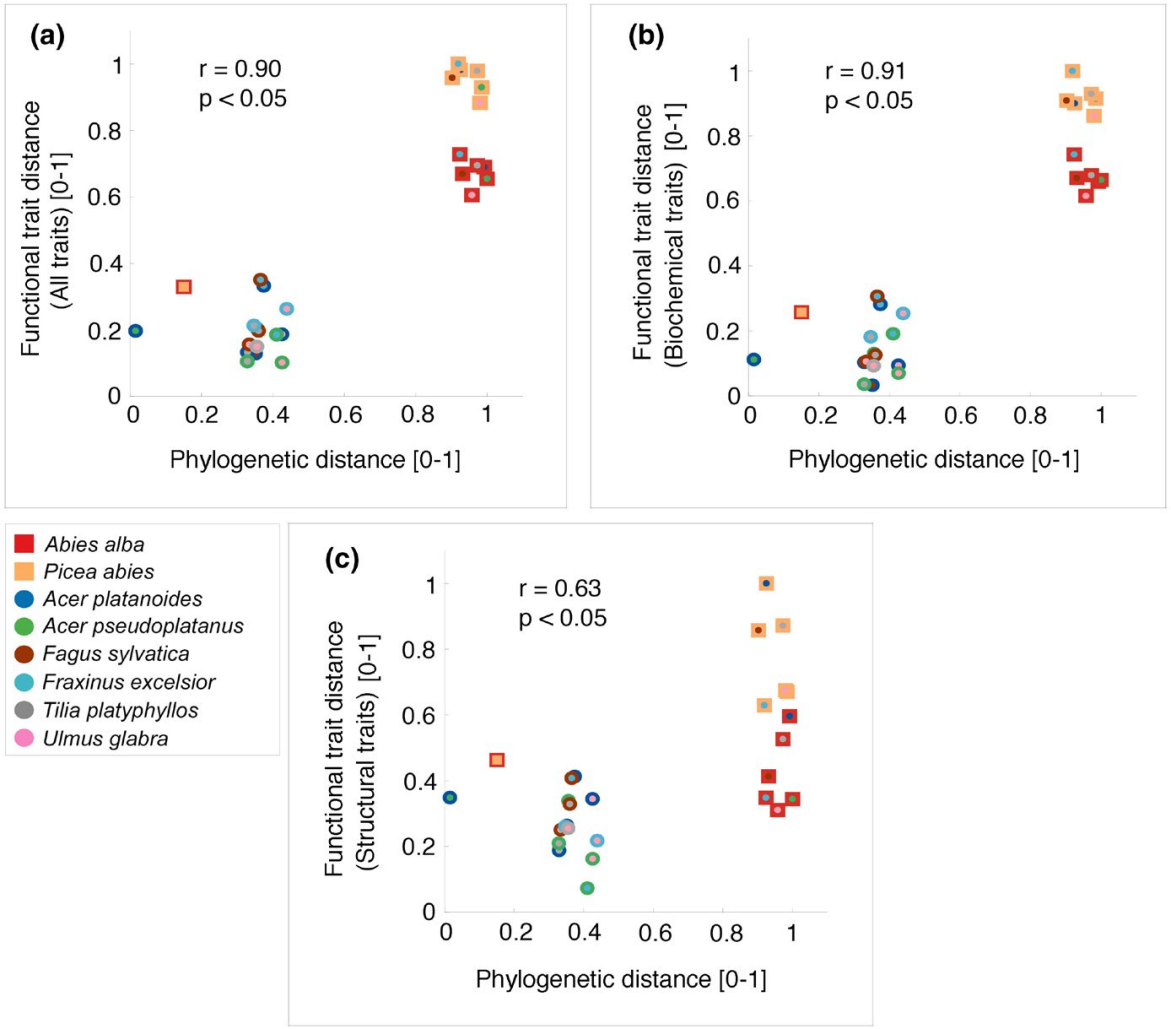
Phylogenetic distances versus trait distances between the eight common species at the study site. Trait distances were calculated using (a) all six traits, (b) the three biochemical traits, and (c) the three architectural traits. Squares and dots represent conifers and angiosperms, respectively, and different colors represent different species (legend). All distances have been rescaled using a linear transformation to a value range of 0–1

Between‐species trait distances were positively related with between‐species phylogenetic distances (*p* < .05; Figure 5a). This was mainly due to the two classes of conifer versus angiosperm species which clearly differed from each other regarding traits and phylogeny (0.9 < phylogenetic distance < 1). The phylogenetically most similar species also had short trait distances as seen by the clusters of *Ac. platanoides* and *Ac. pseudoplatanus* (phylogenetic distance < 0.1), *Ab. alba* and *P. abies* (phylogenetic distance < 0.2), and the cluster formed by the four remaining angiosperm species (*Fa. sylvatica*, *Fr. excelsior*, *Tilia platyphyllos,* and *U. glabra*; 0.3 < phylogenetic distance < 0.5).

In accordance with the ANOVA results, distances calculated with the three biochemical traits (*r* = .91, *p* < .05; Figure 5b) were more closely correlated with phylogenetic distances than distances calculated with the three architectural traits (*r* = .63, *p* < .05; Figure 5c).

**FIGURE 6:**
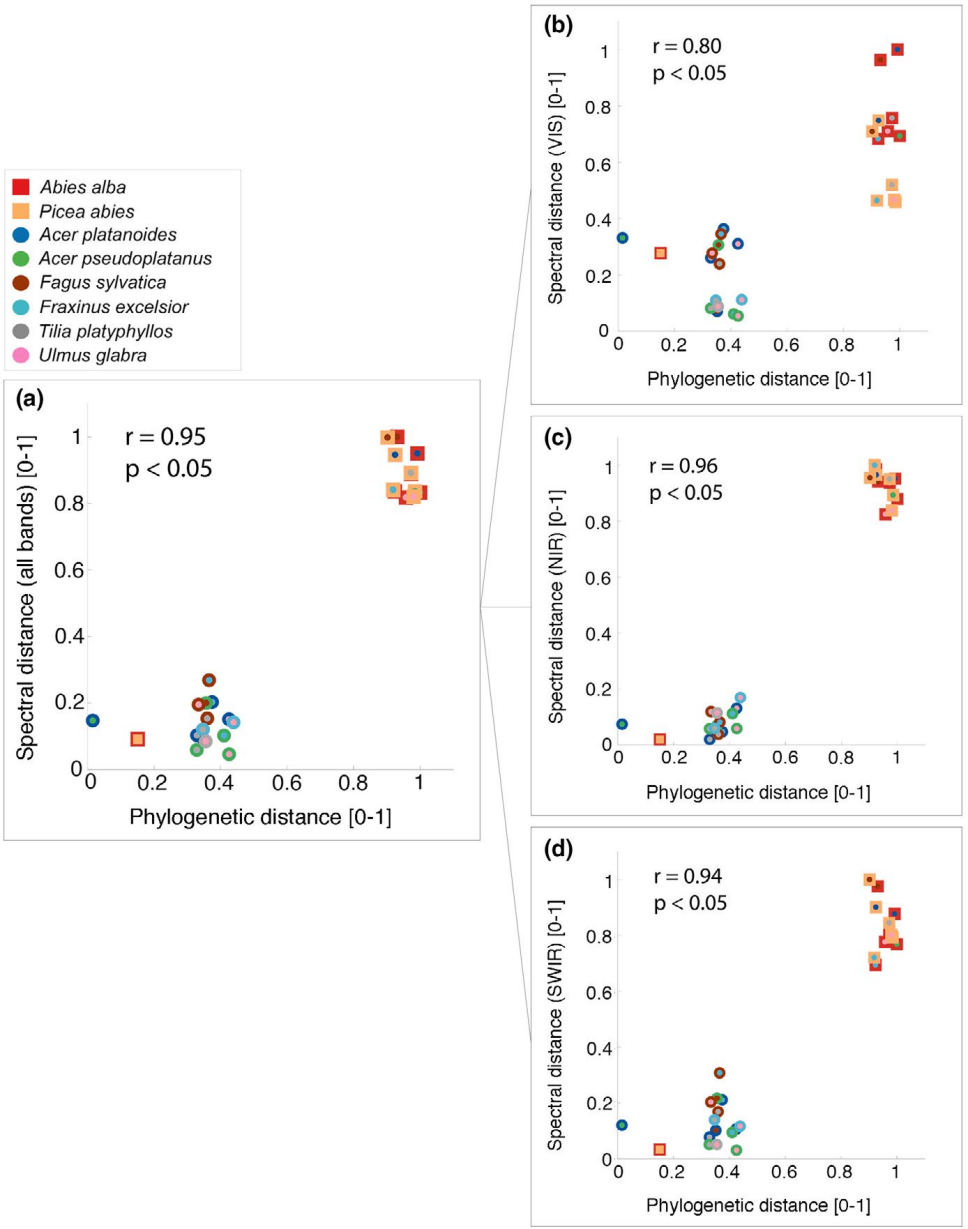
Phylogenetic distance versus Spectral distance. Spectral distance has been calculated using: (a) all spectral bands and using the spectral bands corresponding to the three main spectral regions (b) VIS, (c) NIR, and (d) SWIR. Functional trait distance has been calculated by using groups of: (a) all six traits and (b) three biochemical traits, and (c) three architectural traits. Each species' mean spectral distance is plotted against each other, for each of the four cases (a–d), on the phylogenetic distance space. Squares and dots represent conifers and angiosperms, respectively, and different colors represent different species (legend). All distances have been rescaled using a linear transformation to a value range of 0–1

We also calculated between‐species distances directly with the raw spectral data instead of the derived trait data. Using all spectral bands, there was a close correlation between spectral distances and phylogenetic distances (*r* = .95, *p* < .05; Figure 6a). As for the correlation with trait distances, the distances between the two classes of conifer versus angiosperm species were driving this overall positive correlation. Among three main regions of the spectrum, visible (VIS: 400–700 nm; Figure 6b), near infrared (NIR: 700–1,300 nm; Figure 6c), and short‐wave infrared (SWIR: 1,300–2,500 nm; Figure 6d), the NIR region was the one that best captured the phylogenetic separation between taxa (*r* = .96, *p* < .05), followed by the SWIR (*r* = .94, *p* < .05) and the VIS (*r* = .80, *p* < .05) regions. This corresponds to the large amount of variation in LWC explained by the explanatory term class in the ANOVA. This trait is derived from two spectral bands in the NIR region (Formula A3 from Appendix 1).

### 4.3 Within‐species trait variation

**FIGURE 7:**
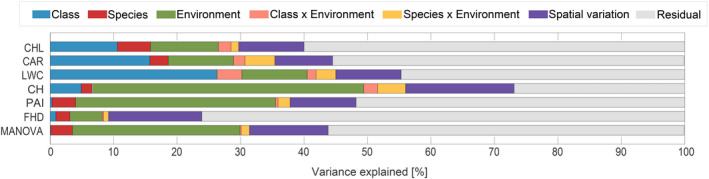
Variance partitioning for all six traits. Amount of variance explained by taxonomic groups (class and species in blue and red, respectively), environmental variables (including soil type, soil depth, volume of coarse grain in brown tones, PAR, aspect, elevation, slope, curvature, presence of herbs, and presence of understory) in green, interactions of the two (in orange and yellow, respectively), spatial variation (90‐ and 40‐m grid cells) in purple and residual variation in gray

Taxonomic groups represent between‐species trait variation whereas the rest of the trait variation among tree individuals must be due to environmental variables or genetic variation within species. Although our study was carried out at a single site, environmental gradients across this site were large and an accordingly large amount of within‐species, among‐individual trait variation could be explained by environmental variables (Appendices 8, 9, 10, 11, 12, 13, 14). We therefore asked in our third question in Section 1 how environmental variation influences trait variation and how these environmental responses may vary among taxonomic units. Taking only significant environmental variables into consideration, they together explained 26.4% of the total trait variation in the MANOVA including all six traits (Appendix 14). Environmental variation more strongly affected architectural traits (42.8% in CH, 31.5% in PAI, and 5.1% in FHD) than biochemical traits (10.7% in CHL, 10.3% in CAR, and 10.3% in LWC). PAR and altitude had the strongest influence on trait variation, and for PAR, there were also the strongest differences among species as reflected by class × environment and species × environment interactions, which together typically explained about 3% of trait variation (Appendices 8, 9, 10, 11, 12, 13). As expected (Khurana & McLaren, 1982), PAI strongly increased with PAR, in particular in the dominant species *Fa. sylvatica* but also *Ac. platanoides* (Appendix 18). Canopy height also increased with PAR, but more strongly in conifers than in angiosperms (Appendix 20). CHL decreased with PAR in all species except *Fa. sylvatica* and *Fr. excelsior* (Appendix 15) and CAR increased with PAR in angiosperms (Appendix 16). The remaining trait variation among individuals was partly explained by spatial variation at the scale of 90‐ or 40‐m grid cells (between 9.1% and 17.1% of trait variation), leaving 27.0% (CH) to 76.1% (FHD) unexplained residual variation (Appendices 8, 9, 10, 11, 12, 13). The results of the ANOVAs are summarized in Figure 7 for all six traits. Using the fitting sequence taxonomic variables > environmental variables > interaction > spatial grids, we can see that all of these contribute to trait variation among individual trees but environmental variation is more important for architectural traits whereas taxonomic variation and species × environmental interactions are relatively more important for biochemical traits.

## 5 DISCUSSION

Our goal in the present study was to test whether plant functional traits could be assessed at individual level using remotely sensed (RS) spectral and lidar data, thus making it possible to analyze variation between and within taxonomic units with complete spatial coverage at a single site with tools commonly used for field‐collected data (He et al., 2009; Li et al., 2017), where individuals are a natural sampling unit. Previous studies used pixel‐level RS data and thus focused on community‐level analysis of trait variation (Asner, Martin, Tupayachi, et al., 2014; Schneider et al., 2017), because even within small pixels multiple species may be present or single individuals may occur in multiple pixels.

We could demonstrate that in a temperate mixed conifer‐angiosperm forest with 13 tree species, of which eight were common enough to be further investigated, it was possible to delineate the individual crowns of all canopy trees (*n* = 1,307). Six functional traits, three biochemical and three architectural ones, could be extracted for each individual from its spectral and lidar data. General linear models followed by ANOVAs showed that variation among taxonomic units was related to the deep phylogenetic split between gymnosperms and angiosperms, but significant variation was also found among species within the two classes for all six traits. Taxonomic variation was larger for biochemical than architectural traits, indicating that the former holds greater promise to detect phylogenetic and taxonomic variation among individuals in the face of even larger environmental variation. However, even by combining information from all traits less than half of all individuals could be correctly assigned to species. Further improvements may be gained by using the total spectral “fingerprint”, as indicated by the stronger correlation between spectral rather than trait distances with phylogenetic distances, or additional foliar traits that can be derived from imaging spectroscopy. Important plant functional traits of the leaf economic spectrum (Wright et al., 2004), such as leaf nitrogen, leaf mass per area, or phosphorus, can be derived from the reflectance spectrum in the visible to short‐wave infrared wavelength range using statistical models (Wang et al., 2020), if corresponding field data are available to train the models.

Among the strongest environmental drivers of trait variation were PAR and elevation, but soil variables (especially amount of rocks) were also important. Compared with taxonomic and environmental variables, interactions of these contributed less to variation. Nevertheless, species responded significantly differently to environmental gradients, even in direction (i.e., positive vs. negative), which may contribute to their coexistence at the spatially highly variable test site.

Overall trait variation in a species‐diverse plant community at a single site is always related to contributions from both taxonomic and environmental variation. These contributions can only be separated if traits are assessed at individual level. The separation of taxonomic and environmental contributions offers a basis to further analyze community assembly and ecosystem functioning, but will require additional information about temporal changes (Levine et al., 2017) and community‐level variables such as primary productivity (Durán et al., 2019). For example, the considerable trait variation we found within species implies that this allows for within‐species functional diversity, potentially increasing primary productivity (Crutsinger, 2006; Latzel et al., 2013). However, to reach an even higher overall functional diversity, multiple species and multiple higher taxonomic units should be present at a single site. Mapping traits at individual level allows to combine both, within‐ and between‐species functional diversity, as we show here. Thus, high values of LWC in the southern area of our study site were due to all species having higher LWC at low elevation as well as to a greater abundance of conifers with higher average LWC. Higher LWC in conifers than in broad‐leaved species has previously been reported by Huber et al. (2008) and recently by Robakowski et al. (2020), who also suggest that due to their water‐relations broad‐leaved species are more sensitive to reduced water supply. We suggest that needles can store more water due to their larger surface area to volume ratio compared with broader, flatter leaves for a mixed temperate forest in Switzerland.

Although separating taxonomic from environmental variation requires individual‐level data, assigning individuals to taxonomic units using spectral data still remains a challenge, especially for closely related species such as the various broad‐leaved, deciduous species in the studied temperate forest. Since phylogenies can give information on the evolutionary history of species, which have been marked by a set of genetic changes over time, phylogenetic relatedness between individuals should give an idea about the similarity of their functional traits (niche conservatism; Ackerly, 2003; Silvertown et al., 2006). When we compared the value of different traits for taxonomic separation, biochemical traits generally were more closely related to phylogenetic and taxonomic differences than were architectural traits, maybe because the first are more directly related to underlying genetic differences among individuals than the second. This suggests that, by extension, such traits might also be more promising for detecting genetic variation within species as a next step in using spectral information in trait‐based ecology (Czyz et al., 2020). Furthermore, such traits that are closely associated with genetic variation could likely be derived from the spectral reflectance, as indicated by the stronger correlation between spectral distances and phylogenetic distances than between the distances calculated with the selected six traits and the phylogenetic distances. That is, reflectance spectra should capture more phenotypic properties than the six traits assessed here. It has been estimated that at least 30 dimensions should be represented in the spectral data with the range used in the present study (Thompson et al., 2017). Additional discriminatory power might be achieved with multiple temporal acquisitions of spectral data (Czyz et al., 2020), because phenotypic properties are not constantly expressed through time, for example, some might be expressed early and others late in the growing season.

Substantial trait variation among individuals within species could be related to environmental gradients across the site, to which the species responded in parallel (main effects of environmental variables in tables from Appendices 8, 9, 10, 11, 12, 13, 14) and also with some significant variation (interactions in tables from Appendices 8, 9, 10, 11, 12, 13, 14). Such plasticity (Bradshaw, 1965; Sultan, 2000) allows a single species to perform well over a range of environmental conditions, thus affecting both its own performance as potentially also the performance of the entire plant community (Bongers et al., 2020; Roscher et al., 2018). Typically, traits that are particularly important for plant growth express adaptive plasticity, whereas other traits may be more constantly expressed across different environments, for example, plant reproductive traits (Schmid, 1992). In our case, the two architectural traits PAI and CH showed very strong plasticity (more than 30% of total variation), increasing with PAR, whereas the three biochemical traits showed weaker responses (around 10% of total variation) that also varied more among species. Spatial and residuals variation among individuals within species was large for all traits, which is typical for analysis of field‐measured individual trait data (Bongers et al., 2020; Li et al., 2017). Together spatial and residual variation could reflect biotic or abiotic microenvironmental variation (Pearson & Dawson, 2003), genetic variation, or different tree developmental stages such as different tree age (Funk et al., 2017). We tested the latter by using CH as an age‐related covariate in ANOVAs for the other five traits; however, this contributed little explanatory power, and corresponding results are therefore not presented. In comparison with pixel‐level data, these results are not surprising, because residuals in the latter case are not accounting for all variation among individuals but rather some of the individual variation still can reside within single pixel. Being variation among individuals, the spatial and residual variation that we found must have had biological reasons, for example, different genotypes, developmental stages, microenvironmental conditions (Alvarez et al., 2013; Kobal et al., 2015; Saremi et al., 2014), biotic interactions, or other features that can be unique for each individual tree (Flood et al., 2011). This unexplained variation between individual trees was largest by far for the architectural trait FHD, suggesting that this complex trait describing the vertical distribution of foliage of individual trees is the least consistent across taxonomic units and environmental gradients and largely depends on the local abiotic and biotic conditions of individuals.

Regarding total community functional trait variation, two main statements can be made when the study is contextualized in temperate forests and at local scales: the six functional traits used in this study present both substantial between‐ and within‐species variation, with biochemical traits showing relatively more taxonomic and architectural traits more environmental variation. These differences between architectural and biochemical traits suggest a high environmental plasticity of tree canopy architecture, which could allow individuals of the same species to better cope with local environmental conditions. These results were obtained for a single‐site study using a complete sample of individuals. It is conceivable that multisite field studies in which not all individuals per site are sampled yield biased representations of within‐species trait variation, for example, if individuals with particular trait values are less likely to be included. In this context, extending our approach using RS data to multiple sites holds the promise to assess total community functional trait variation without bias and to correlate this functional variation with taxonomic and environmental variation across larger gradients (de Bello et al., 2011; Kenzo et al., 2006; Ustin & Gamon, 2010; Waring & Pitman, 1985).

## 6 CONCLUSION

We conclude that with high‐resolution RS data it is possible to delineate individual‐tree crowns within a forest and thus assess functional traits derived from RS data at the individual level. With this precondition fulfilled, it is then possible to apply tools commonly used in field‐based trait ecology to partition trait variation among individuals into taxonomic and potentially even genetic variation, environmental variation, and interactions between the two. This partitioning of trait variation can help us to better understand factors shaping the spatial structure of tree communities (Cavender‐Bares et al., 2006) and thus can be used as a basis for further studies of relationships between functional diversity and community assembly or ecosystem functioning (Durán et al., 2019; Schweiger et al., 2018). For example, trait variation can be related to coexistence mechanisms (such as competitive exclusion leading to niche differentiation and increased phylogenetic dispersion among species; see e.g., Allan et al., 2013; Valladares et al., 2015) or environmental filtering reducing taxonomic variation and phylogenetic dispersion (Thonicke et al., 2020). Incorporating individual‐based trait information into biodiversity dynamics models could also improve predicting responses of vegetation to environmental change at various spatial scales. Furthermore, individual‐level trait information could be incorporated into dynamic vegetation models to predict more accurately the potential effects of functional diversity on forest productivity and other ecosystem functions.

## CONFLICT OF INTEREST

The authors declare no conflicts of interest.

## AUTHOR CONTRIBUTIONS

**Carla Guillén‐Escribà:** Conceptualization (equal); Data curation (lead); Formal analysis (lead); Investigation (lead); Methodology (equal); Validation (equal); Visualization (lead); Writing—original draft (lead); Writing—review and editing (equal). **Fabian D. Schneider:** Data curation (lead); Formal analysis (lead); Investigation (equal); Methodology (equal); Validation (equal); Visualization (equal); Writing—original draft (equal); Writing—review and editing (equal). **Bernhard Schmid:** Conceptualization (equal); Formal analysis (lead); Investigation (equal); Methodology (equal); Supervision (equal); Visualization (equal); Writing—original draft (lead); Writing—review and editing (equal). **Andrew Tedder:** Data curation (equal); Formal analysis (equal); Investigation (supporting); Visualization (equal); Writing—original draft (equal). **Felix Morsdorf:** Conceptualization (equal); Data curation (equal); Formal analysis (equal); Investigation (equal); Supervision (equal); Writing—original draft (supporting); Writing—review and editing (supporting). **Reinhard Furrer:** Formal analysis (supporting); Methodology (supporting); Visualization (supporting). **Andreas Hueni:** Data curation (equal); Writing—original draft (equal). **Pascal A. Niklaus:** Conceptualization (equal); Formal analysis (supporting); Methodology (equal); Supervision (equal). **Michael E. Schaepman:** Conceptualization (lead); Funding acquisition (lead); Methodology (equal); Project administration (lead); Resources (lead); Supervision (equal); Writing—review and editing (equal).

## Data Availability

Data are deposited in the Dryad Digital Repository: https://doi.org/10.5061/dryad.k98sf7m68.

## APPENDIX 1

### Calculations of biochemical traits

Following Schneider et al. (2017), pigment relative content was calculated for each individual tree using the formula from the three‐band model of Gitelson et al. (2006):

(A1) $$\text{CHL}=\left(\frac{1}{R_{540-560}}-\frac{1}{R_{760-800}}\right)\cdot R_{760-800},$$

(A2) $$\text{CAR}=\left(\frac{1}{R_{510-520}}-\frac{1}{R_{560-570}}\right)\cdot R_{760-800},$$

where *R_i_*_ −_ *_j_* is the mean reflectance of the spectral range of *i* to *j* nanometers

The three‐band model of Gitelson et al. (2006) was developed for noninvasive estimation of chlorophyll, carotenoids, and anthocyanins. As anthocyanin content in leaves is very low during summer months (Hughes et al., 2005) (when our AIS data were acquired), we decided to select relative LWC as the third biochemical trait of this study since it is a commonly used indicator of plant hydration status. Relative LWC has an important ecological relevance as water deficit of the leaves can affect plant functioning by decreasing photosynthetic potential (Tezara et al., 1999) and by extension growth. The spectral band combinations used for LWC retrieval were the following:

(A3) $$\text{LWC}=1-\frac{R_{1,193}}{R_{1,126}},$$

where *R_i_* is the reflectance at *i* nanometers. All three biochemical traits were linearly normalized from 0 to 1.

## APPENDIX 2

### Calculations of architectural traits

Following Schneider et al. (2017), CH was retrieved by using the distance between the highest laser return and the corresponding ground point for each individual tree. PAI, defined as half of the total area of leaves and woody materials per unit ground area (Chen & Black, 1992), is a more accurate term than LAI for ALS‐derived canopy density measures by also including woody parts of the tree. Since most of the studies deriving LAIs from ALS data are in fact referring to PAI (Korhonen & Morsdorf, 2014), our results are directly comparable with them. To retrieve PAI, we used a formula (see Schneider et al., 2014) developed by Solberg et al. (2009) and modified by Fleck et al. (2012), which includes canopy and ground echoes in the vertical column of 2 × 2 m grid cells. FHD, which is a measure of canopy layering complexity, was derived for canopy layers of 5 m and retrieved by using the following formula (MacArthur & MacArthur, 1961):

(A4) $$\text{FHD}=-\sum_{i}p_{i}\cdot \log_{e}\left(p_{i}\right),$$

where *p_i_* is the proportion of the total foliage lying at the *i*th canopy layer. Units for CH are meters, for PAI m^2^/m^2^ and FHD is a unitless index.

## APPENDIX 3

### Validation of biochemical functional traits

To evaluate the performance of the three spectral indices at the leaf level, two optical methods were used to determine the leaf optical properties and the relative chlorophyll content of 50 beech individuals at the test site using stratified random sampling. The sampling strategy sought to optimize sampling time and costs by focusing on a representative set of the most dominant species at the test site to cover the full range (including extreme values) of architectural traits and environmental gradients that could impact functional trait diversity within species. The totality of beech trees was spread in nine groups (from the combination of low/medium/high slope and low/medium/high tree height). From each of the groups, nine trees were randomly selected (four groups had less than nine trees) obtaining a stratified random sample of *n* = 50. Three leaves from three different branches from the top of each of the 50 crowns were collected by tree climbers. Reflectance and transmittance were measured at three different positions on each of the leaves using a field spectroradiometer (ASD FieldSpec Pro.) attached to an external ASD single‐leaf clip. A white and black spectralon background in the leaf clip was used to derive both reflectance and transmittance following the method described in Kukenbrink et al. (2019) and in Kükenbrink et al. (2021), but only used reflectance measured on a black background in this study. Relative chlorophyll content was measured for the same leaves with a chlorophyll meter (SPAD 502). After assessing the performance of the indices at the leaf level, leaf‐level chlorophyll estimations were compared with relative chlorophyll content of modeled canopy spectra (see Schneider et al., 2017), see figure below.

*Note*. SPAD measurements from 50 *Fagus sylvatica* trees randomly selected at our test site compared with corresponding chlorophyll estimations from leaf optical properties.

## APPENDIX 4

### Canopy pigment and leaf water content estimations for each individual at our test site compared with 2010 data from the same trees (Schneider et al., 2017)

*Note*. Scatter plots of: (a) relative CHL, (b) CAR content, and (c) relative LWC of all trees from the test site with data acquired in 2010 versus 2016.

## APPENDIX 5

### Histograms of frequency distribution for all biochemical and architectural traits

*Note*. (a) Histogram for the three biochemical traits (CAR, relative carotenoid content; CHL, relative chlorophyll content; LWC, relative water content) and (b) Histogram for the three architectural traits (CH, canopy height; FHD, foliage‐height diversity; PAI, plant area index). All traits have been rescaled to a value range of 0–1.

## APPENDIX 6

### Pearson's correlation coefficient matrix for all six traits (CHL, CAR, LWC, PAI, CH, and FHD) and four environmental variables (PAR, slope, curvature, and elevation)

*Note*. Scatter plots and distributions histograms are represented in dark and light blue, respectively, and correlation coefficients in red.

## APPENDIX 7

### Sequence information of two commonly sequenced genes, *matK*, and *rbcl* for each of the 26 species included in the phylogenetic tree

**Table:**

Species name	*matK*	*rbcL*
*Abies alba*	HQ619823.1	HQ619762.1
*Acer campestre*	HE970664.1	HE963303.1
*Acer platanoides*	KU549962.1	FN689356.1
*Acer pseudoplatanus*	KU550031.1	KJ204286.1
*Alnus glutinosa*	HQ600562.1	FN689372.1
*Betula pendula*	FJ011829.1	GU373385.1
*Carpinus betulus*	JN894261.1	HF567847.1
*Cotoneaster integerrimus*	JQ390969.1	JQ391316.1
*Daphne mezereum*	JN894977.1	KF613076.1
*Elaeagnus angustifolia*	KP089052.1	KP088577.1
*Fagus sylvatica*	JN895534.1	FN689362.1
*Fraxinus excelsior*	HM171489.1	FJ862056.1
*Malus sylvestris*	JN894106.1	JN890877.1
*Morus nigra*	GU145558.1	JX571868.1
*Picea abies*	HE966963.1	HE963590.1
*Pinus sylvestris*	KP089957.1	EU677093.1
*Prunus spinosa*	HE966978.1	FN689384.1
*Pyrus communis*	JN895841.1	JN892983.1
*Quercus petraea*	JN894728.1	HE963626.1
*Rhamnus cathartica*	KJ593087.1	KJ593659.1
*Rhus typhina*	KM212097.1	KJ841514.1
*Salix viminalis*	JN894736.1	AJ849577.1
*Sorbus aria*	JN894460.1	GQ248697.1
*Spiraea alba*	KJ593109.1	KJ841598.1
*Tilia platyphyllos*	KP089345.1	JN891178.1
*Ulmus glabra*	JN895720.1	JN893088.1

Source: GenBank.

## APPENDIX 8

### Analysis of variance for leaf chlorophyll content

**Table:**

Source of variation	*df*	*SS*	*MS*	*F*	*p*	%*SS*
Class	1	119	118.9	211.77	<.0000	10.6
Species	6	59	9.8	17.4	<.0001	5.2
Soil	2	63	31.7	56.5	<.0001	10.7
PAR	1	5	5.1	9.12	.0026	
asp	1	5	4.9	8.68	.0033	
alt	1	11	11	19.67	<.0001	
cur	1	36	36.4	64.77	<.0001	
Class × PAR	1	22	21.9	38.99	<.0000	2.0
Species × PAR	6	14	2.3	4.01	.00056	1.2
G90	14	51	3.6	6.48	<.0001	10.3
G40	48	65	1.4	2.4	<.0001	
Residuals	1,202	675	0.6	*R* ^2^	.400	60.0

Note

Explanatory terms (source of variation) were fitted sequentially as single terms or groups. Terms within groups are not separated by horizontal lines. Percentages of sum of squares (%*SS*) are increments of multiple *R* ^2^*100 and combined for groups. Thus, tables from Appendices 8, 9, 10, 11, 12, 13, 14 are comparable in that %*SS* are given for class and species as taxonomic terms, all environmental terms combined, the interactions of taxonomic and environmental terms, and the spatial variation reflected by the two grids G90 and G40 (see figure from Appendix 22). At least one environmental term is always included in the interactions (cf. table from Appendix 14), but additional nonsignificant terms are excluded from the model.

Abbreviations: *df*, degrees of freedom; *F*, variance ratio; *MS*, mean squares; *p*, probability of type‐I error.

## APPENDIX 9

### Analysis of variance for leaf carotenoid content

**Table:**

Source of variation	*df*	*SS*	*MS*	*F*	*p*	%*SS*
Class	1	170	170.3	334.18	<.0001	15.7
Species	6	32	5.3	10.5	<.0001	2.9
Soil	2	6	3.1	6.1	.0023	10.3
soilRocks	1	25	25.3	49.7	<.0001	
PAR	1	45	44.9	88.1	<.0001	
alt	1	16	15.8	31.0	<.0001	
cur	1	20	20.3	39.8	<.0001	
Class × Soil	2	1	0.4	0.7	.4903	1.8
Class × soilRocks	1	2	1.9	3.8	.0517	
Class × PAR	1	13	13	25.52	<.0001	
Class × alt	1	4	4.5	8.8	.0031	
Species × Soil	9	14	1.6	3.15	.0009	4.7
Species × soilRocks	5	9	1.8	3.59	.0032	
Species × PAR	6	13	2.2	4.34	.0002	
Species × alt	6	15	2.5	4.97	<.0001	
G90	14	33	2.4	4.64	<.0001	9.1
G40	48	66	1.4	2.71	<.0001	
Residuals	1,178	600	0.5	*R* ^2^	.446	55.4

Note

For explanation see Appendix 8.

## APPENDIX 10

### Analysis of variance for leaf water content

**Table:**

Source of variation	*df*	*SS*	*MS*	*F*	*p*	%*SS*
Class	1	0.131	0.131	676.08	<.0001	26.3
Species	6	0.0194	0.0032	16.7	<.0001	3.9
Soil	2	0.0069	0.0035	17.84	<.0001	10.3
soilRocks	1	0.0103	0.0103	53.22	<.0001	
soilDepth	1	0.0019	0.0019	9.72	.0019	
PAR	1	0.0034	0.0034	17.53	<.0001	
alt	1	0.0174	0.0174	89.91	<.0001	
slo	1	0.001	0.001	5.08	.0245	
cur	1	0.0031	0.0031	16.02	<.0001	
her	7	0.0033	0.0005	2.42	.0185	
und	6	0.0038	0.0006	3.25	.0036	
Class × PAR	1	0	0	0.04	.8437	1.4
Class × alt	1	0.0022	0.0022	11.12	.0009	
Class × cur	1	0.0041	0.0041	21.02	<.0001	
Class × her	5	0.0005	0.0001	0.55	.7349	
Species × PAR	6	0.0038	0.0006	3.26	.0035	3.1
Species × alt	6	0.0021	0.0003	1.77	.1020	
Species × cur	6	0.0017	0.0003	1.44	.1954	
Species × her	21	0.008	0.0004	1.96	.0060	
G90	14	0.0158	0.0011	5.81	<.0001	10.3
G40	48	0.0354	0.0007	3.81	<.0001	
Residuals	1,147	0.2223	0.0002	*R* ^2^	.553	44.7

Note

For explanation see Appendix 8.

## APPENDIX 11

### Analysis of variance for plant area index

**Table:**

Source of variation	*df*	*SS*	*MS*	*F*	*p*	%*SS*
Class	1	6	6	5.65	.0176	0.3
Species	6	86	14	14.26	<.0001	3.7
Soil	2	12	6	6.01	.0025	31.5
PAR	1	692	692	691.85	<.0001	
her	7	17	2	2.45	.0172	
Class × Soil	2	1	0	0.42	.6583	0.4
Class × PAR	1	7	7	7.24	.0072	
Species × Soil	9	23	3	2.56	.0065	1.9
Species × PAR	6	21	4	3.58	.0016	
G90	14	72	5	5.11	<.0001	10.4
G40	48	166	3	3.46	<.0001	
Residuals	1,187	1,187	1	*R* ^2^	.482	51.8

Note

For explanation see Appendix 8.

## APPENDIX 12

### Analysis of variance for foliage‐height diversity

**Table:**

Source of variation	*df*	*SS*	*MS*	*F*	*p*	%*SS*
Class	1	0.5	0.498	13.43	.0003	0.9
Species	6	1.3	0.215	5.79	<.0001	2.2
Soil	2	0.3	0.157	4.22	.0149	5.1
soilRocks	1	0.6	0.637	17.18	<.0001	
asp	1	0.2	0.185	4.98	.0259	
alt	1	1.4	1.382	37.29	<.0001	
cur	1	0.5	0.534	14.4	.0002	
Class × cur	1	0.1	0.086	2.31	.1290	0.2
Species × cur	6	0.5	0.079	2.13	.0476	0.8
G90	14	3.1	0.225	6.06	<.0001	14.7
G40	48	5.5	0.114	3.07	<.0001	
Residuals	1,202	44.6	0.037	*R* ^2^	.239	76.1

Note

For explanation see Appendix 8.

## APPENDIX 13

### Analysis of variance for canopy height

**Table:**

Source of variation	*df*	*SS*	*MS*	*F*	*p*	%*SS*
Class	1	1,232	1,232	202.83	<.0001	4.9
Species	6	411	68	11.28	<.0001	1.7
Soil	2	965	483	79.49	<.0001	42.8
soilRocks	1	946	946	155.74	<.0001	
soilDepth	1	626	626	103.08	<.0001	
PAR	1	2,270	2,270	373.88	<.0001	
asp	1	210	210	34.55	<.0001	
alt	1	3,762	3,762	619.56	<.0001	
slo	1	87	87	14.35	.00016	
cur	1	1,239	1,239	204.1	<.0001	
her	7	299	43	7.02	<.0001	
und	6	284	47	7.8	<.0001	
Class × Soil	2	16	8	1.28	.27752	2.2
Class × soilRocks	1	18	18	3.04	.08143	
Class × soilDepth	1	0	0	0.03	.8143	
Class × PAR	1	366	366	60.33	<.0001	
Class × her	5	86	17	2.82	.01533	
Class × und	3	68	23	3.75	.01074	
Species × Soil	9	172	19	3.15	.00093	4.4
Species × soilRocks	5	180	36	5.92	<.0001	
Species × soilDepth	5	126	25	4.14	.00099	
Species × PAR	6	139	23	3.83	.00088	
Species × her	21	333	16	2.61	<.0001	
Species × und	24	137	6	0.94	.54496	
G90	14	1876	134	22.07	<.0001	17.1
G40	48	2,391	50	8.2	<.0001	
Residuals	1,110	6,740	6	*R* ^2^	.730	27.0

Note

For explanation see Appendix 8.

## APPENDIX 14

### MANOVA of all six traits

**Table:**

Source of variation	*df*	*SS*	*MS*	*F*	*p*	%*SS*
Class	1	1	1.00	0.36	.5523	0.1
Species	6	56	9.33	3.34	<.0001	3.4
soilRocks	1	15	15.00	5.37	<.0001	26.4
soilDepth	1	7	7.00	2.51	.1199	
PAR	1	218	218.00	78.09	<.0001	
asp	1	7	7.00	2.51	.1199	
alt	1	165	165.00	59.10	<.0001	
und	7	16	2.29	0.82	.5765	
Class × PAR	1	3	3.00	1.07	.3051	0.2
Species × PAR	6	22	3.67	1.31	.2695	1.3
G90	14	67	4.79	1.71	<.0001	12.4
G40	48	134	2.79	1.00	<.0001	
Residuals	1,196	910	0.76	*R* ^2^	.439	56.1

Note

For explanation see Appendix 8.

## APPENDIX 15

### Leaf chlorophyll content (arbitrary units) of individuals of eight tree species as a function of light availability (PAR, W m^−2^ day^−1^)

*Note*. Simple regression lines are shown, for significance of overall slope, species differences and interactions see Appendix 8.

## APPENDIX 16

### Leaf carotenoid content (arbitrary units) of individuals of eight tree species as a function of light availability (PAR, W m^−2^ day^−1^)

*Note*. Simple regression lines are shown, for significance of overall slope, species differences and interactions see Appendix 9.

## APPENDIX 17

### Leaf water content (arbitrary units) of individuals of eight tree species as a function of light availability (PAR, W m^−2^ day^−1^)

*Note*. Simple regression lines are shown, for significance of overall slope, species differences and interactions see Appendix 10.

## APPENDIX 18

### Plant area index (unitless) of individuals of eight tree species as a function of light availability (PAR, W m^−2^ day^−1^)

*Note*. Simple regression lines are shown, for significance of overall slope, species differences and interactions see Appendix 11.

## APPENDIX 19

### Foliage height diversity (arbitrary units) of individuals of eight tree species as a function of altitude (m above sea level)

*Note*. Simple regression lines are shown, for significance of overall slope, species differences and interactions see Appendix 12.

## APPENDIX 20

### Canopy height (m) of individuals of eight tree species as a function of light availability (PAR, W m^−2^ day^−1^)

*Note*. Simple regression lines are shown, for significance of overall slope, species differences and interactions see Appendix 13.

## APPENDIX 21

### Discriminant analysis for individuals of eight tree species

*Note*. The first two classification axis are shown (LD1 and LD2).

## APPENDIX 22

### Spatial distribution of the environmental variables at the test site

*Note*. Slope, elevation, and PAR are continuous variables while curvature, aspect, soil type, soil depth, presence of coarse gravel, and grids 40 and 90 m are categorical variables.
